# The Influence of Eating at Home on Dietary Diversity and Airway Inflammation in Portuguese School-Aged Children

**DOI:** 10.3390/ijerph18052646

**Published:** 2021-03-05

**Authors:** Francisca de Castro-Mendes, Pedro Cunha, Inês Paciência, João Cavaleiro Rufo, Mariana Farraia, Diana Silva, Patrícia Padrão, Luís Delgado, André Moreira, Pedro Moreira

**Affiliations:** 1 Serviço de Imunologia Básica e Clínica, Departamento de Patologia, Faculdade de Medicina da Universidade do Porto, 4200-319 Porto, Portugal; inespaciencia@gmail.com (I.P.); mariana.farraia@gmail.com (M.F.); disolha@gmail.com (D.S.); ldelgado@med.up.pt (L.D.); andremoreira@med.up.pt (A.M.); 2 EPIUnit—Instituto de Saúde Pública, Universidade do Porto, 4050-091 Porto, Portugal; jcrufo@gmail.com (J.C.R.); patriciapadrao@fcna.up.pt (P.P.); pedromoreira@fcna.up.pt (P.M.); 3 Faculdade de Ciências da Nutrição e Alimentação da Universidade do Porto, 4150-177 Porto, Portugal; pedrocunha27@hotmail.com; 4 Serviço de Imunoalergologia, Centro Hospitalar São João, 4200-319 Porto, Portugal

**Keywords:** airway inflammation, children, diet, dietary quality, dietary diversity, eating at home, eNO

## Abstract

Considering the negative impact of a lack of dietary diversity on children’s nutritional status, we aimed to describe dietary variety according to eating at home frequency and assessed its association with respiratory outcomes in school-aged children. This cross-sectional study included 590 children (49% girls) aged 7 to 12 years from 20 public schools located in city of Porto, Portugal. Daily frequency of eating at home groups were calculated and dietary diversity was calculated using a 10-food group score from a 24 h recall questionnaire. Spirometry and exhaled nitric oxide levels (eNO; <35 and ≥35 ppb) were assessed. The comparison of diet diversity according to the groups was performed by ANOVA and ANCOVA. The association between dietary diversity and respiratory outcomes was examined using regression models. In multivariate analysis, children in the highest group of eating at home episodes (≥4 occasions) obtained the lowest dietary diversity mean score, while the lowest group (<2) had the highest mean score (*p*-value 0.026). After adjustment for confounders, higher diet diversity (≥5 food groups) significantly decreased the odds of having an eNO ≥35. Diet diversity might decrease the chance of airway inflammation among children. However, having more eating episodes at home could be a barrier to a more diverse diet.

## 1. Introduction

In parallel with the increasing incidence of allergic diseases, there has been a change in diet, which is framed in the western lifestyle pattern [1]. This western dietary pattern is characterized by a high intake of processed meats, refined grains, and sugary foods, and low intake of plant-based foods [2], which might compromise the supply of important dietary components with anti-oxidant and anti-inflammatory properties involved in protection against asthma development [3]. Among these dietary components, the lower intake of fruits and vegetables, which are important sources of dietary fibre, micronutrients, and phytochemicals that participate in antioxidant and anti-inflammatory metabolic activities, seems to be a potential contributor for the risk of asthma development [4]. However, dietary components are not consumed alone and may exhibit synergic effects within the food and between foods when consumed together [5], and asthma is an heterogeneous disease that results from several risk factors [6].

Considering the negative impact of the lack of dietary diversity on child nutritional status [7], indicators of diet variety are attractive as a measure of nutritional adequacy [8,9]. Furthermore, diet diversity may be relatively simple to measure, and could expand the limitation of assessing the effects of diets from single dietary components [8,9], while considering the interactions of the matrixes from a variety of foods on health [10,11]. Several observational studies have evaluated the dietary quality through the assessment of the Mediterranean diet, which is characterized not only by a higher consumption of fruits, vegetables, and wholegrain cereals, but also by a greater variety of these foods [12]. Higher adherence to the Mediterranean diet has been associated with less occurrence of asthma [13], less asthma symptoms [14,15], and asthma control [16]. On the other hand, in adults, higher healthy eating dietary scores, including the Alternate Healthy Eating Index 2010, were associated with a lower asthma symptom score [17] and asthma symptoms improvement in longitudinal analysis with a 7-year follow-up period [18]. In children, a higher diet quality score decreased the odds of having asthma [19].

In accordance with the western lifestyle, eating episodes outside home also increased, which might be an explanation for a poorer diet quality [20]. It was found that children who regularly consumed takeaway meals at home had an higher daily energy intake [21]. However, eating outside the home is not always related with poorer eating quality, and the results are mixed. Results from a Portuguese birth cohort showed that preschool children having more meals in preschools consumed more vegetables, fruits, cereals, and fish and presented a better dietary adequacy [22], and other studies have also documented a higher nutritional quality of school meals compared with meals from home [23,24]. More recently, Calixto Andrade et al. evaluated the association between eating out and ultra-processed food consumption, founding a positive association between the consumption of ultra-processed foods and eating out of home, when compared to individuals eating at home [25]. Similarly, in a representative sample from UK children, those following a pattern of eating out of home had a higher consumption of ultra-processed foods compared to those following a pattern related to eating at school with friends [26].

Children spend most of their day out of home, so it is plausible that dietary diversity and nutritional intake might be impacted according the foods obtained at home and outside the home, particularly at school [23], consequently affecting diseases such as asthma and its related outcomes. One of the measures used to assess diet diversity in children is the Minimum Dietary Diversity for Women (MDD-W) of reproductive age assessment, which was originally developed to evaluate diet diversity through micronutrient adequacy at the population-level from non-pregnant women of reproductive age [27]. It was also shown that a higher MDD-W was modestly associated with a lower risk of ischemic heart disease [28]. In children aged 4 to 8 years, higher MDD-W was associated with overall micronutrient adequacy [29]. However, there are no previous studies evaluating the differences of MDD-W score between eating locations and its association with respiratory and allergic outcomes. Therefore, we aimed to describe diet diversity according to eating at home frequency, and to assess the association between diet diversity and asthma, lung function, and airway inflammation as well as respiratory and asthma symptoms in school-aged children.

## 2. Materials and Methods

### 2.1. Study Design and Participants

This cross-sectional study included 590 participants aged 7 to 12 years, who attended the 3rd and 4th grades at 71 classrooms from 20 public schools located in the city of Porto, Portugal [30]. From the 1602 children who were invited to participate, those who did not provide informed consent (*n* = 686), refused to perform clinical procedures (*n* = 58), or had incomplete nutritional data were not included (*n* = 268). Significant differences between included and not included participants were found for forced expiratory volume in the first second (FEV1) and forced expiratory flow middle portion of forced vital capacity (FEF25-75) reversibility and positive bronchodilation (*p* < 0.05, Table S1).

Written consent was obtained from each child’s legal guardian or parent. The Ethics Committee of the University Hospital São João approved the study. Every procedure was carried out in accordance with Helsinki Declaration.

### 2.2. Diet and Minimum Dietary Diversity for Women (MDD-W) at Reproductive Age Assessment

The participants were asked about their diet through a single 24 h recall questionnaire. According to a standard procedure, this questionnaire allowed the detailed collection of data from all the foods and beverages consumed in the previous 24 h, including the quantity, respective food brands and cooking methods [31]. These data were recorded per eating occasions that included information about the place and time of consumption. Eating occasions were defined based on any occasion when a food or beverage is consumed with at least 30 min of interval and providing a minimum of 50 kcal of energy [32]. The eating occasions performed outside of home included all the meals that were not eaten at home, including other homes, such as those of friends, grandparents, or other relatives, at schools, restaurants, or cafes, “on the go” (e.g., car/bus or stress) and leisure places (e.g., sports club). Daily frequency of eating at home groups were defined based on the number of eating occasions performed at home: G1, less (<) than 2; G2, equal to 2; G3, equal to 3; and G4, more (≥) than 4. Total energy intake (kcal) was assessed through the software Food Processor^®^ (ESHA Research, Salem, OR, USA), which encompasses databases of Portuguese nutritional food-composition and was used as a continuous variable.

Dietary diversity was assessed through the Minimum Dietary Diversity for Women (MDD-W) at reproductive age assessment [33]. Ten food groups were defined according the guidelines for MDD-W in which ingredients in mixed dishes or unmixed foods and beverages consumed in quantities ≥15 g were matched [33]: grains, white roots and tubers, and plantains (“starchy staples”, including all types of breads, pasta or potatoes); pulses (beans, peas, and lentils); nuts and seeds (mostly tree nuts but, groundnut as well as nut and seed “butters”); dairy (almost all liquid and solid dairy products from cows, goats, buffalo, sheep or camels); meat, poultry, and fish (meats, organ meats, poultry and other birds and fresh and dried fish and seafood/shellfish); eggs; dark green leafy vegetables (medium green leaves, including Chinese cabbage, romaine and bibb lettuce, broccoli or spinach along with darker greens); other vitamin A-rich fruits and vegetables (namely ripe mango and papaya, passion fruit, apricot and several types of melons, as well as, carrot, pumpkin and deep yellow- or orange-fleshed squash); other vegetables (all the vegetables not considered as dark green leafy vegetables or as other vitamin A-rich vegetables, namely tomato or cucumber); and other fruits (all the fruits not included as vitamin A-rich fruits, namely sweet white bananas, apple, or kiwi). Each consumed group received 1 point and were counted to create the MDD-W diversity score, which range between 0 and 10. A dichotomous indicator was also calculated based on whether or not participants have consumed at least five out of ten defined foods [33]. We also considered other food groups of concern as suggested in this score [33], namely “other oils and fats” (e.g., butter, margarine and “shortening”), “savory and fried snacks” (e.g., doughnuts/fried dough), “sweets” (e.g., candy, chocolates and cakes), “sugar-sweetened beverages” (e.g., “juice drinks”, chocolate drinks and sweet tea), and “red and/or processed meat”. Although these groups were not included to calculate the MDD-W, we counted them to create the “concerning food score”.

### 2.3. Current Respiratory and Asthma Symptoms Assessment

The legal guardian answered a questionnaire based on the International Study of Asthma and Allergies in Childhood (ISAAC), which posed questions about social, demographic, and behavioral information and included questions about respiratory/allergic health and respiratory symptoms [34].

Asthma symptoms in the previous year, which includes wheezing and cough symptoms, were defined based on a positive answer to the question “did your child have wheezing or whistling in the chest in the past 12 months?”, and “did your child suffer coughing at night in the last 12 months?” or “did your child suffer coughing more than three months in the last year?”.

Current respiratory symptoms were defined based on a positive answer to the question “during the past 3 months, has your child had any of the following symptoms?”: “irritative cough” and “breathing difficulties”.

### 2.4. Spirometry and Asthma Assessment

Spirometry with bronchodilation was performed. Lung function was evaluated before and 15 min after the inhalation of 400 μg of salbutamol following the official American Thoracic Society/ The European Respiratory Society (ATS/ERS)guidelines [35]. Positive bronchodilation (+BD) was defined by at least a 12% and over 200 mL increase in FEV1. Self-report of asthma diagnosed by a physician was defined based on a positive answer to the question “has your child ever been diagnosed with asthma by a physician?”.

As previously described [36], three different definitions of asthma were considered: (i) ever: self-reported medical diagnosis; (ii) medical diagnosis with asthma symptoms or +BD—self-reported medical diagnosis with reported symptoms (wheezing, dyspnea or dry cough) occurring in the past 12 months or +BD; and (iii) medical diagnosis and under asthma treatment—self-reported medical diagnosis and currently under anti-asthma medication.

### 2.5. Fractional Exhaled Nitric Oxide (eNO) Assessment

Fractional exhaled nitric oxide was measured through a NObreath analyzer (Bedfont Scientific Ltd., Rochester, Kent, UK) to assess airway inflammation. The results were expressed as parts per billion (ppb) and stratified according to the official ATS guidelines for children [37], which were dichotomized according to the cut point equal or above (≥) 35 ppb as increased levels of eNO.

### 2.6. Other Covariates

Allergic sensitization was evaluated through skin-prick-tests (SPT), which were performed on children’s forearms using a QuickTestTM (Panatrex Inc., Placentia, California, USA applicator), which included house dust mite, mix of weeds (*Urtica dioica*, *Plantago lanceolate and Artemisia vulgaris*), mix of grasses *(Agrostis stolonifera*, *Anthoxathum odoratum*, *Dactylis glomerata*, *Lolium perenne*, *Arrhenatherium elatius*, *Festuca rubra*, *Poa pratensis*, *Holcus lanatus*, *Phleum pratense*, *Secale cereal*), cat dander, dog dander, and Alternaria alternata, negative control (extracts dilutant), and a positive control consisting of histamine at 10 mg/mL (Hall Allergy, Netherlands). Results were read after 15 min. Atopy was defined based on a positive SPT (wheal ≥3 mm diameter) to at least one of the allergens.

Self-reported medical diagnosis of food allergy (FA) diagnosis was defined based on a positive answer to the following two questions “has your child ever been allergic to any food?” and “was the allergy diagnosed by the doctor?”.

Weight was measured using a digital scale (Tanita™ BC-418 Segmental Body Analyzer) and recorded in kilograms (kg). Height was assessed with a portable stadiometer and recorded in centimeters (cm). Weight and height were used to calculate body mass index (BMI), computed as kg per square meters (kg/m^2^), and classified according to age- and sex-specific percentiles defined by the US Centers for Disease Control and Prevention (CDC) [38].

Parental’ education level is presented as the number of successfully completed years of formal schooling of the parent at the higher education level and categorized into three classes: ≤9 years; ≥10 years and ≤12 years; and >12 years. Maternal smoking during pregnancy was obtained from a positive answer to the question “did the mother smoke during pregnancy?”. Exposure to tobacco at home was defined by a positive answer to the question “is your child exposed to tobacco smoke at home?”. Child nutritional supplementation was assessed through a positive question “has your child taken nutritional supplements (vitamins/minerals) in the past year?”.

### 2.7. Statistical Analyses

Continuous variables are expressed as median (25th–75th percentile), while categorical variables are presented as percentages. To determine differences between groups of daily frequency of eating at home, the Kruskal-Wallis test for continuous variables and the chi-squared test for categorical variables were performed. To describe the differences between daily frequency of eating at home groups, we calculated the number and percentage of children consuming foods from each food group.

The comparison of mean MDD-W score according to groups of daily frequency of eating at home was performed using ANOVA. ANCOVA was used to adjust for potential confounders: age, sex, atopy, self-reported medical diagnosis of FA, BMI categories, parental education, child nutritional supplementation, number of eating occasions, and energy intake. Bonferroni correction for multiple comparisons was applied.

Linear regression models (β) were used to analyze the association between lung function and the MDD-W indicator. Similarly, logistic regression models (odds ratio; OR) were used to investigate the association between diet diversity and the increased levels of eNO, +BD, and asthma definitions, as well as asthma and current respiratory symptoms. These regressions were adjusted in three consecutive models as follows: crude model—main effect; model 1—age, sex, atopy, self-reported medical diagnosis of FA, BMI categories, parental education, maternal smoking during pregnancy, tobacco smoke at home, child nutritional supplementation, and daily frequency of eating at home groups; and model 2—model 1, concerning food score and total energy intake. Asthma defined by medical diagnosis with asthma symptoms or +BD was additionally considered in the linear regression model. The interaction of groups of daily frequency of eating at home groups on the association with each outcome was examined by a product term added into fully adjusted models. No significant interactions were detected (*p*-value > 0.05)

A 0.05 level of significance and 95% confidence interval (CI) were considered. SPSS statistical package software v26.0 (IBM, Armonk, NY, USA) was used to carry out all the analyses.

## 3. Results

The proportion of participants eating 5 or more food groups (G1 = 80.30%; G2 = 79.40; G3 = 67.40; and G4 = 58.60, *p*-value = 0.001, Table 1) and the total energy (kcal) intake (G1 = 2067.80; G2 = 2228.00; G3 = 2246.84; and G4 = 2465.77, *p*-value < 0.001) was significantly different among daily frequency of eating at home groups.

All participants consumed starchy staple foods, and nearly all consumed dairy, meat, poultry, and fish, other vegetables, and other fruits (Table 2). Children who consumed >4 eating occasions at home had the lowest consumption of other vegetables (Q1 = 90.20%, Q2 = 84.80%, Q3 = 76.30%, and Q4 = 58.60%; *p*-value = 0.001, Table 2) and other fruits (Q1 = 82.90%, Q2 = 82.40%, Q3 = 75.60%, and Q4 = 56.90%; *p*-value < 0.001, Table 2). These children had also the highest consumption of sweets (Q1 = 66.84%, Q2 = 79.41%, Q3 = 73.33%, and Q4 = 84.48%; *p*-value = 0.009).

Crude and adjusted mean of MDD-W score according to groups of daily frequency of eating at home are presented in Table 3. Children who recorded <2 eating occasions at home (G1 = 5.37, 95% CI 5.20; 5.55) and those who recorded ≥4 (G4 = 4.74, 95% CI 0.16; 4.42) presented the highest difference in MDD-W score (*p*-value = 0.002). In multivariate analysis, G1 was characterized by a significantly higher MDD-W score compared to the other groups (G1 = 4.98, 95% CI 4.46; 5.49; G2 = 4.48, 95% CI 4.35; 5.42; G3 = 4.75, 95% CI 4.20; 5.31; and G4 = 4.24, 95% CI 3.63; 4.84, *p*-value = 0.005). No statistically significant differences were observed in MDD-W score between G3 and the other groups.

In fully adjusted models (model 2), children who consumed at least 5 food groups had decreased odds of having increased levels of eNO (OR = 0.46, 95% CI 0.24; 0.89, *p*-value = 0.020, Table 4). No significant associations were found between the MDD-W indicator with positive bronchodilation or asthma definitions (Table 4). However, regarding respiratory symptoms in the previous 12 months, in adjusted models those with higher diet diversity consumption were more likely report coughs (OR = 2.48, 95% CI 1.05; 5.87, *p*-value = 0.038). No significant associations were found between diet diversity and lung function (Table S2).

## 4. Discussion

This cross-sectional study showed that children having less eating occasions at home had higher diet diversity compared to those who have more eating occasions at home. Furthermore, a greater diet diversity consumption was associated with lower eNO, as well as higher odds of having a cough in the previous year.

In our study, the location of each eating occasion was determined based on where the food was consumed. Although previous studies applied the same definition [22,25], others used different criteria, including the place where the meal was prepared [39,40]. This use of different definitions makes comparisons between studies difficult. Nevertheless, it was previously demonstrated that in children aged between 6 to 12 years, diet quality (measured through the Healthy Eating Index) was negatively associated with meals away from home, while no association was found with meals from school [39]. Similarly, among participants aged 11 to 18 years, the overall diet quality score was significantly higher among low and moderate consumers (B = 2.8, 95% CI 1.0, 4.6%; *p* < 0.01 and B = 3.4%, 95% CI 1.7, 5.0%, *p* < 0.01, respectively) compared with frequent consumers of meals out [41]. In addition, a study conducted in restaurants and shopping centers from Portugal found that children’s menus often include nutritionally poor food and had low availability of vegetables and fruits [42].

Although the diet in Portugal is frequently associated with the Mediterranean pattern, results from national studies showed a high intake of fruits and vegetables but also a high consumption of energy-dense foods such as sugar sweetened beverages and snacks [43]. Nevertheless, the results from the present study showed that children consuming more meals at home had lower diet diversity, which was accompanied by a higher total energy and sweets intake. A possible explanation might be the fact that among the 68% of children who had meals at schools, 65% reported that they consumed at least half of their meals at school (data not shown), which might have positively impacted the diet diversity of those who consumed less meals at home. In accordance, results from the Portuguese birth cohort Generation XXI found that the mean dietary adequacy index was higher for children in the “pre-school” group (18.3, 95% CI 18.1, 18.4) when compared to other groups, including “home” (17.6, 95% CI 17.1, 17.7, *p* < 0.001) [22]. In addition, Vepsäläinen et al., showed that schools from Portugal had more availability of healthy foods compared to empty-calorie foods [44]. It was also showed that there was a low household adherence to the Mediterranean dietary pattern in all Portuguese regions [45]. Results from the National Diet and Nutrition Survey Rolling Program, which included children aged 4 to 10 years, showed that the mean overall intake of fruits and vegetables was higher when meals were prepared at schools rather than prepared at home (66.2 ± 2.5 vs. 59.3 ± 2.8, *p* = 0.06) [40].

Our results revealed a negative association between dietary diversity and increased levels of eNO. Although the role of dietary components on eNO levels has been previously assessed, the majority of published results come from studies conducted in patients with asthma. Nevertheless, it was shown that among children with asthma, the consumption of butter, which has high content of saturated fatty acids (SFA), was positively correlated with FeNO levels (r = 0.29, *p* = 0.001) [46]. This correlation was negative for the consumption of salad (r = −0.20, *p* = 0.025) [46]. Similarly, results from a cross-sectional study of adult patients with asthma revealed a positive association between n-6:n-3 polyunsaturated fatty acids, and monounsaturated fatty acids: SFA ratio with eNO (β = 0.05, 95% CI 0.02, 0.09 and β = 0.64, 95% CI 0.08, 1.20, respectively) [47]. Children with moderate to severe asthma whose diet was supplemented with nutritional/dietary supplements (resveratrol, folic acid, magnesium, zinc, vitamin D, among others, for 1 month), had significantly reduced FeNO levels (baseline = 19.0 vs. after = 11.0, *p* = 0.03), while the non-supplemented group did not presented significant differences (18. vs. 16.0, *p* = 0.13) [48].

On the other hand, there are studies showing no associations between dietary components and eNO levels. Chambers and Ayres showed that among healthy participants, the oral supplementation with ascorbic acid did not change the eNO levels [49]. In adults with allergies, there were no significant associations between the serum levels of antioxidant dietary compounds, such as vitamins C, E, b-carotene, and selenium, and eNO levels [49]. More recently, in a double-blind, placebo-controlled, randomized trial of 3 days, adult patients with asthma were assigned to the broccoli sprouts group, in which the intervention implicated the consumption of 100 g of broccoli sprouts daily or to the placebo group, which requires the consumption of 100 g of alfalfa sprouts, there were no significant reductions of eNO levels [50]. The difference in the results might be explained through the fact that an individuals’ diet is composed of different dietary compounds that are consumed together and may exhibit synergic effects within and between foods [5]. In addition, the number of participants was low, which might compromise the power to detect significant differences. Altogether, these results highlight the importance of studying the entire diet to consider the complexity of food matrixes, as we did in the present study.

Endogenous NO is important in airway function regulation, being involved in respiratory diseases such as asthma [51]. The evaluation of eNO has been used to assess airway inflammation, and although it is correlated with eosinophilic airway inflammation, it is not present in all patients with asthma [52]. In fact, in the present study, among children with higher eNO (*n* = 75), only 18 have asthma defined by self-reported medical diagnosis with reported symptoms (wheezing, dyspnea, or dry cough) occurring in the past 12 months or positive bronchodilation (data not shown). Moreover, our group previously showed that eNO had very low diagnostic accuracy for asthma [53]. Nonetheless, the mechanisms that led its production, either to be a mediator of cell damage or a mechanism of defense under pro-oxidant environments, are not completely understood [46]. Cardinale et al., hypothesized that pro-oxidant environments with high levels of pro-inflammatory cytokines might increase the activity from the inducible NO synthase enzyme, which will protect the airways from oxidative stress damage [14]. Considering the previous hypothesis, it is also expected that a higher dietary diversity, with higher nutrient adequacy and dietary quality, will provide several dietary components with anti-oxidant and anti-inflammatory properties that might decrease the production of pro-inflammatory cytokines [12], consequently decreasing the NO production.

Contrarily to what we were expecting, the results from our study showed a positive association between diet diversity and cough in the previous 12 months. In fact, a systematic review concluded that higher adherence to the Mediterranean diet was negatively associated with asthma symptoms in children [15]. More recently, another systematic review emphasized a potential negative association between dietary diversity and wheeze and asthma [54]. A possible explanation might be the cross-sectional design of the study, in which these associations might have resulted from a reverse causation, where the consumption of dietary diversity was modified because of a previous diagnosis of allergic or respiratory condition. This study has a few limitations. Its cross-sectional design does not allow establishing causal relationships between dietary diversity consumption and the respiratory outcomes. However, it is unlikely that an inverse causal inference occurred, since we assessed important lifestyle variables which might be related with both diet and respiratory outcomes [55], including total energy intake, food allergy, atopy, and body mass categories that, together with other confounders, were taken into consideration in the association between diet diversity and respiratory outcomes. We collected a single 24 h recall questionnaire, although multiple recalls are better to report habitual intake [56]. In addition, it is designed to evaluate short-term intake and did not account for seasonality differences. Moreover, the reported intake in the previous 24 h might not represent a typical day. Nonetheless, 24 h recall questionnaires were collected by nutritionists and trained interviewers who applied photos and food models, which helped the children to quantify portion sizes without misreporting, avoiding influencing children’s responses [57]. Furthermore, we collected detailed information about cooking methods, ingredients used in mixed dishes, brands of food and beverages consumed, and portion sizes. In this regard, the 24 h recall questionnaire seems to be useful to estimate children’s current diet since it avoids the introduction of errors associated with memory and knowledge about food [58], even knowing that dietary data might be affected by a recall bias. Additionally, recalling the most recent foods and beverages consumed in the previous 24 h might be easier for children. Analyzing diet diversity consumption by groups organized by daily eating at home frequency just have to account for the number of meals consumed at home, which might influence the error of the association of certain food groups to specific eating occasions. However, groups allow comparison between groups with the highest and lowest number of eating occasions at home, being less sensitive to the effects of outliers than continuous variables [59]. Confounders were selected based on both knowledge of their relationship with diet and with the outcomes evaluated and evidence from previous literature, although residual confounding may still exist. Lastly, the generalization of the results should be performed with caution, considering that the research is restricted to Portugal. We also acknowledge some strengths to the present study. To our knowledge, this is the first study evaluating the MDD-W score among groups of daily eating at home frequency in school-aged children and its association with asthma and its related outcomes. Diet was classified according to MDD-W score, used to evaluate the overall diet diversity [33], and validated as a proxy measure for assessing micronutrient adequacy at the population-level [27]. In addition, this score was shown to be better for the characterization of seasonal variability and micronutrient adequacy among children between 4 to 8 years of age when compared to the 7-food group score [29]. We considered different respiratory outcomes and asthma definitions, including objective measures obtained from spirometry with bronchodilation and ISAAC self-reported responses [53]. Lastly, the data collected was detailed and performed by the same research team, allowing a relatively unbiased estimation of outcomes prevalence.

## 5. Conclusions

Our study showed that a higher diet diversity consumption was negatively associated with airway inflammation. In addition, having more eating episodes at home might be a possible barrier to better diet diversity. Strategies aimed to promote diets with more diversity of food groups, providing adequacy in micronutrients, should be prioritized.

## Figures and Tables

**Table 1:** Characteristics of the participants according to daily frequency of eating at home groups.

Participants Characteristics	G1, *n* = 193 (32.70)	G2, *n* = 204 (34.60)	G3, *n* = 135 (22.90)	G4, *n* = 58 (9.80)	Total, *n* = 590	*p*-Value
Age (years)	9.00 (8.00–9.00)	9.00 (8.00–9.00)	9.00 (8.00–9.00)	9.00 (8.00–9.00)	9.00 (8.00–9.00)	0.967
Female sex, *n* (%)	100 (51.80)	91 (44.60)	66 (48.90)	32 (55.20)	289 (49.0)	0.381
Atopy ^1^, *n* (%)	60 (31.40)	76 (37.40)	49 (36.80)	19 (32.80)	204 (34.90)	0.586
Self-reported medical diagnosis of FA, *n* (%)	13 (6.70)	13 (6.40)	5 (3.70)	5 (8.60)	36 (6.10)	0.540
BMI categories ^2^, *n* (%)						0.346
Underweight	7 (3.60)	12 (5.90)	4 (3.00)	5 (8.60)	20 (4.70)	
Normal weight	129 (66.80)	151 (74.00)	95 (70.40)	42 (72.40)	417 (70.7)	
Overweight	34 (17.60)	23 (11.30)	19 (14.10)	5 (8.60)	81 (13.70)	
Obese	23 (11.90)	18 (8.80)	17 (12.60)	6 (10.3)	64 (10.80)	
Parental education ^3^, *n* (%)						0.620
≤9	48 (33.80)	60 (36.10)	40 (36.00)	21 (44.70)	169 (36.30)	
≥10 and ≤12	39 (27.50)	52 (31.30)	38 (34.20)	13 (27.70)	142 (30.50)	
>12	55 (38.70)	54 (32.50)	33 (29.70)	13 (27.70)	155 (33.30)	
Maternal smoking during pregnancy, *n* (%)	47 (26.40)	50 (26.20)	31 (25.00)	14 (26.90)	142 (26.10)	0.991
Tobacco at home, *n* (%)	56 (34.10)	74 (43.00)	40 (34.80)	21 (41.20)	191 (38.0)	0.308
Child nutritional supplementation, *n* (%)	22 (13.30)	26 (14.20)	18 (14.90)	13 (25.00)	79 (15.10)	0.210
Increased levels of eNO ^4^, *n* (%)	17 (8.90)	32 (15.80)	18 (13.50)	8 (13.80)	75 (12.80)	0.230
Positive bronchodilation ^5^, *n* (%)	13 (6.70)	6 (2.90)	9 (6.70)	4 (6.90)	32 (5.40)	0.290
Asthma definitions, *n* (%)						
Ever	11 (6.00)	21 (10.70)	5 (3.80)	4 (7.10)	41 (7.20)	0.097
Medical diagnosis with asthma symptoms or +BD	19 (9.80)	20 (9.80)	9 (6.70)	6 (10.30)	54 (9.20)	0.725
Medical diagnosis and under asthma treatment	9 (4.70)	16 (7.80)	4 (3.00)	4 (6.90)	33 (5.60)	0.238
Lung function						
FVC, L	1.90 (1.67–2.16)	1.90 (1.71–2.14)	1.89 (1.69–2.19)	1.81 (1.64–2.06)	1.89 (1.69–2.14)	0.338
FVC, % predicted	105.91 (96.94–116.77)	103.09 (94.55–112.10)	104.39 (95.90–114.44)	109.68 (99.67; 118.20)	104.66 (95.73–115.29)	0.075
FEV1, L	1.77 (1.57–1.97)	1.75 (1.60–1.98)	1.77 (1.59–1.95)	1.67 (1.55–1.86)	1.75 (1.58–1.95)	0.446
FEV1, % predicted	102.10 (91.46–112.67)	99.09 (90.34–108.95)	99.5 (93.52–108.28)	103.73 (95.21–114.38)	100.76 (91.65–110.20)	0.202
FEF25-75, L/s	2.36 (1.92–2.67)	2.27 (1.94–2.61)	2.23 (1.97–2.62)	2.28 (2.03–2.59)	2.28 (1.95–2.63)	0.890
FEF25-75, % predicted	93.80 (83.16–108.92)	91.32 (81.76–109.42)	94.65 (83.00–109.54)	95.85 (81.88–104.79)	93.76 (82.41–108.60)	0.954
FEV1 reversibility, %	3.80 (0.00; 7.45)	2.98 (0.00–6.06)	3.66 (0.00–6.71)	2.68 (0.53–6.76)	3.47 (0.00–6.70)	0.863
FEF25-75 reversibility, %	10.04 (2.09–18.94)	10.27 (1.03.19.35)	10.81 (2.73–20.59)	10.27 2.62–16.50)	10.27 (2.09–19.02)	0.875
Asthma symptoms ^6^, *n* (%)						
Wheezing	16 (8.30)	20 (9.80)	9 (6.70)	5 (8.60)	50 (8.50)	0.791
Cough	19 (9.80)	25 (12.30)	17 (12.60)	8 (13.80)	69 (11.70)	0.788
Current respiratory symptoms ^7^, *n* (%)						
Breathing difficulties	18 (10.60)	24 (13.00)	8 (6.60)	4 (7.80)	54 (10.20)	0.311
Irritative cough	47 (27.20)	51 (27.40)	46 (37.70)	11 (21.60)	155 (29.10)	0.096
MDD-W indicator (≥5), *n* (%)	155 (80.3)	162 (79.4)	91 (67.4)	34 (58.60)	442 (74.90)	0.001
Concerning food score	3.00 (2.00; 3.00)	3.00 (2.00; 3.00)	3.00 (2.00; 3.00)	3.00 (2.00; 4.00)	3.00 (2.00; 3.00)	0.142
Total Energy Intake (kcal)	2067.80 (1793.74–2340.08)	2228.00 (1898.18–2515.23)	2246.84 (1886.95–2511.18)	2465.77 (1969.71–2862.35)	2179.14 (1872.71–2487.68)	<0.001

Data are expressed as medians (25th–75th percentile) unless otherwise stated. ^1^: defined by a positive skin prick test to at least one of the allergens; ^2^: according to US Centers for Disease Control and Prevention; ^3^: number of successfully completed years of formal schooling; ^4^: ≥35 ppb; ^5^: defined by at least a 12% and over 200 mL increase in forced expiratory volume in 1 s (FEV1); ^6^: in the previous 12 months; ^7^: in the previous 3 months; FA: food allergy BMI: body mass index; eNO: exhaled nitric oxide; +BD: positive bronchodilation; FVC: forced vital capacity; FEV1: forced expiratory volume in the first second; FEF25-75: forced expiratory flow middle portion of FVC; FEV1 reversibility: forced expiratory volume in the first second after bronchodilation; FEF25-75 reversibility: forced expiratory flow middle portion of FVC after bronchodilation. MDD-W: The Minimum Dietary Diversity for Women of reproductive age assessment.

**Table 2:** Consumption of food groups from the MDD-W according to groups of daily frequency of eating at home.

Food Groups from MDD-W	G1, *n* = 193 (32.70)	G2, *n* = 204 (34.60)	G3, *n*= 135 (22.90)	G4, *n* = 58 (9.80)	Total, *n* = 590	*p*-Value
Grains, white roots and tubers, and plantains	193 (100)	204 (100)	135 (100)	58 (100)	590 (100)	--
Pulses (beans, peas, and lentils)	51 (26.40)	53 (26.00)	34 (25.30)	10 (17.20)	148 (25.10)	0.538
Nuts and seeds	3 (1.60)	6 (2.90)	0 (0.00)	1 (1.70)	10 (1.70)	0.235
Dairy	148 (76.70)	162 (79.40)	110 (81.50)	53 (91.40)	473 (80.20)	0.099
Meat, poultry, and fish	188 (97.40)	200 (98.00)	132 (97.80)	55 (94.80)	575 (97.50)	0.582
Eggs	18 (9.30)	16 (7.80)	10 (7.40)	4 (6.90)	48 (8.10)	0.895
Dark green leafy vegetables	36 (18.70)	46 (22.50)	29 (21.50)	12 (20.70)	123 (20.80)	0.812
Other vitamin A-rich fruits and vegetables	66 (34.20)	65 (31.90)	34 (25.20)	15 (25.90)	180 (30.50)	0.283
Other vegetables	174 (90.20)	173 (84.80)	103 (76.30)	34 (58.60)	484 (82.00)	<0.001
Other fruits	160 (82.90)	168 (82.40)	102 (75.60)	33 (56.90)	463 (78.50)	<0.001
Other oils and fats	116 (60.10)	116 (56.86)	74 (54.81)	37 (63.79)	343 (58.13)	0.611
Savory and fried snacks	15 (7.77)	13 (6.37)	14 (10.37)	5 (8.62)	47 (7.97)	0.612
Sweets	129 (66.84)	162 (79.41)	99 (73.33)	49 (84.48)	439 (74.41)	0.009
Sugar-sweetened beverages	164 (84.97)	180 (88.23)	121 (89.63)	52 (89.66)	517 (87.62)	0.564
Red meat and/or processed	84 (43.52)	93 (45.59)	53 (39.26)	25 (43.10)	255 (43.22)	0.720

MDD-W: The Minimum Dietary Diversity for women of reproductive age assessment; *p*-value was determined by the chi-square test of association between percentage of children consuming a food group and groups of daily frequency of eating at home.

**Table 3:** MDD-W score according to groups of daily eating at home frequency (mean values and 95% confidence intervals).

MDD-W Score	G1	G2	G3	G4	*p*-Value
Crude	5.37 (5.20; 5.55) *	5.36 (5.19; 5.23) *	5.10 (4.89; 5.30)	4.74 (0.16; 4.42)	0.002
Adjusted	4.98 (4.46; 5.49) *	4.88 (4.35; 5.42) *	4.75 (4.20; 5.31)	4.24 (3.63; 4.84)	0.005

MDD-W: The Minimum Dietary Diversity for Women of reproductive age assessment; means adjusted to age, sex, atopy, self-reported medical diagnosis of FA, body mass index categories, parental education, child nutritional supplementation, number of eating occasions and total energy; *: Mean value was significantly different from that for Q4 (<0.05).

**Table 4:** Association between increased levels of eNO, positive bronchodilation, asthma definitions, asthma symptoms in the previous 12 months and current respiratory symptoms in the previous 3 months with MDD-W indicator (odds ratio (OR) and 95% confidence interval (CI)).

Outcomes	Crude Model	*p*-Value	Model 1	*p*-Value	Model 2	*p*-Value
Increased levels of eNO	0.57 (0.34; 0.96)	0.035	0.46 (0.24; 0.88)	0.019	0.46 (0.24; 0.89)	0.020
+BD	0.72 (0.33; 1.56)	0.723	0.57 (0.24; 1.35)	0.203	0.56 (0.24; 1.33)	0.562
Asthma definitions						
Eve	0.83 (0.41; 1.68)	0.832	1.16 (0.44; 3.01)	0.769	1.19 (0.45; 3.13)	0.727
Medical diagnosis with asthma symptoms or +BD	0.95 (0.50; 1.81)	0.881	1.03 (0.47; 2.22)	0.950	1.02 (0.47; 2.21)	0.965
Medical diagnosis and under asthma treatment	0.76 (0.35; 1.63)	0.478	1.17 (0.41; 3.37)	0.770	1.18 (0.41; 3.41)	0.761
Asthma symptoms						
Wheezing	1.07 (0.54; 2.10)	0.853	1.68 (0.66; 4.26)	0.275	1.68 (0.66; 4.27)	0.279
Cough	1.12 (0.62; 2.03)	0.699	2.39 (1.02; 5.60)	0.046	2.48 (1.05; 5.87)	0.038
Current respiratory symptoms						
Breathing difficulties	0.76 (0.41; 1.39)	0.370	0.90 (0.41; 1.96)	0.792	0.89 (0.41; 1.96)	0.782
Irritative cough	1.11 (0.72; 1.71)	0.631	1.57 (0.91; 2.68)	0.103	1.58 (0.92; 2.71)	0.098

MDD-W: The Minimum Dietary Diversity for Women of reproductive age assessment. Increased levels of eNO: fractional exhaled nitric oxide levels ≥35 ppb; +BD: positive bronchodilation defined by at least a 12% and over 200 mL increase in forced expiratory volume in 1 s (FEV1). Ever asthma: self-reported medical diagnosis; medical diagnosis or +BD: self-reported medical diagnosis or positive bronchodilation; medical diagnosis with asthma symptoms or +BD: self-reported medical diagnosis with reported symptoms (wheezing, dyspnea or dry cough) occurring in the past 12 months or positive bronchodilation; medical diagnosis and under asthma treatment: self-reported medical diagnosis and currently under anti-asthma medication; asthma symptoms: symptoms in the previous 12 months; current respiratory symptoms: symptoms in the previous 3 months; crude model: main effects; model 1: age, sex, atopy, self-reported medical diagnosis of FA, body mass index categories, parental education, maternal smoking during pregnancy, tobacco smoke at home, child nutritional supplementation, and groups of daily eating at home frequency; model 2: model 1, concerning food score and total energy intake.

## Data Availability

The data presented in this study are available on reasonable request from the corresponding au-thor. The data are not publicly available due to ethical requirements.

## Supplementary Materials

The following are available online at https://www.mdpi.com/1660-4601/18/5/2646/s1, Table S1: Characteristics of the participants included and non-included, Table S2: Association between lung function and MDD-W indicator (β and 95% CI).
