# Supplementary material for: The Influence of Eating at Home on Dietary Diversity and Airway Inflammation in Portuguese School-Aged Children
Source: Int J Environ Res Public Health. 2021 Mar 5;18(5):2646. doi: 10.3390/ijerph18052646 (PMC7967357; doi:10.3390/ijerph18052646)
Supplement: Supplementary file 1 [file ijerph-18-02646-s001.pdf]

International Journal of
Environmental Research and Public Health

MDPI

Table S1. Characteristics of the participants included and non-included.

Characteristics	Included (*n* = 590)	Excluded (*n* = 268)	Total (*n* = 858)	*p*-value
Asthma symptoms†, *n* (%)				
Wheezing	50 (8.50)	29 (10.82)	79 (9.20)	0.271
Cough	69 (11.70)	29 (10.82)	98 (11.40)	0.709
Current respiratory symptoms‡, *n* (%)				
Breathing difficulties	54 (10.20)	28 (10.45)	82 (10.80)	0.444
Irritative cough	155 (29.10)	77 (28.73)	232 (30.50)	0.203
Lung function, median				
FVC, L	1.89 (1.69–2.14)	1.88 (1.67–2.12)	1.89 (1.69–2.14)	0.509
FVC, % predicted	104.66 (95.73–115.29)	107.38 (96.87–117.44)	105.28 (95.92–115.82)	0.258
FEV1, L	1.75 (1.58–1.95)	1.73 (1.55–1.95)	1.75 (1.58–1.95)	0.538
FEV1, % predicted	100.76 (91.65–110.20)	101.93 (93.86–111.38)	101.26 (92.38–110.96)	0.170
FEF25-75, L/s	2.28 (1.95–2.63)	2.29 (1.89–2.70)	2.28 (1.92–2.65)	0.599
FEF25-75, % predicted	93.76 (82.41–108.60)	95.53 (80.98–114.21)	94.03 (81.99–110.22)	0.427
FEV1 reversibility, %	33.47 (0.00–6.70)	4.27 (0.70–7.79)	3.66 (0.44–7.14)	0.044
FEF25-75 reversibility, %	10.27 (2.09–19.02)	14.19 (5.88–25.00)	11.69 (2.59–20.79)	0.044
Positive bronchodilation§, *n* (%)	32 (5.40)	25 (9.33)	57 (6.60)	0.033
Asthma definitions, *n* (%)				
Ever	41 (7.20)	13 (4.85)	54 (6.50)	0.230
Medical diagnosis with asthma symptoms or +BD	54 (9.20)	26 (9.70)	80 (9.30)	0.798
Medical diagnosis and under asthma treatment	33 (5.60)	13 (4.85)	46 (5.40)	0.655
Increased levels of eNO, *n* (%)	75 (12.80)	25 (9.33)	100 (12.00)	0.258
Atopy ¶, *n* (%)	204 (34.90)	96 (35.83)	300 (35.50)	0.539

Data are expressed as medians (25th–75th percentile) unless otherwise stated; Statistically significant differences between sexes are in bold; †: in the previous 12 months; ‡: in the previous 3 months; §: defined by at least a 12% and over 200mL increase in forced expiratory volume in 1 second (FEV1); ¶: defined by a positive skin prick test to at least one of the allergens; FVC: forced vital capacity; FEV1: forced expiratory volume in the first second; FEF25-75: forced expiratory flow middle portion of FVC; FEV1 reversibility: forced expiratory volume in the first second after bronchodilation; FEF25-75 reversibility: forced expiratory flow middle portion of FVC after bronchodilation.

Table S2. Association between lung function and MDD-W indicator (β and 95% CI).

Characteristics	Crude Model	*p*-value	Model 1	*p*-value	Model 2	*p*-value
FVC	−0.02 (−0.09; 0.05)	0.537	9.00×10^{-3} (−0.07; 0.08)	0.822	9.00×10^{-3} (−0.07; 0.09)	0.823
FVC (% predicted)	0.60 (−2.71; 3.90)	0.723	−1.06 (−4.54; 2.42)	0.550	−1.18 (−4.66; 2.31)	0.507
FEV1	−0.01 (−0.07; 0.05)	0.689	0.02 (−0.05; 0.08)	0.638	0.02 (−0.05; 0.08)	0.633
FEV1 (% predicted)	0.39 (−2.66; 3.44)	0.801	−1.30 (−4.45; 1.86)	0.420	−1.41 (−4.57; 1.74)	0.379
FEF25-75	-7.00×10^{-3} (−0.12; 0.11)	0.899	0.05 (−0.08; 0.18)	0.486	0.05 (−0.09; 0.18)	0.495
FEF25-75 (% predicted)	0.04 (−4.79; 4.87)	0.987	−1.84 (−7.41; 3.74)	0.518	−1.90 (.7.48; 3.69)	0.505
FEV1 reversibility	−0.05 (−1.35; 1.25)	0.941	−0.99 (−2.40; 0.41)	0.166	−1.01 (−2.42; 0.40)	0.160
FEF25-75 reversibility	0.74 (−2.56; 4.05)	0.659	−0.59 (−4.38; 3.21)	0.761	−0.51 (−4.31; 3.29)	0.792

FVC: forced vital capacity; FEV1: forced expiratory volume in the first second; FEF25-75: forced expiratory flow middle portion of FVC; FEV1 reversibility: forced expiratory volume in the first second after bronchodilation; FEF25-75 reversibility: forced expiratory flow middle portion of FVC after bronchodilation; Crude model: main effects; Model 1: age, sex, atopy, self-reported medical diagnosis of FA, body mass index categories, parental education, maternal smoking during pregnancy, tobacco smoke at home, child nutritional supplementation, quartiles of daily eating at home frequency and asthma defined by self-reported medical diagnosis with reported symptoms (wheezing, dyspnea or dry cough) occurring in the past 12 months or positive bronchodilation; Model 2: model 1, concerning food score and total energy intake.
